# Titanium elastic nailing in femoral diaphyseal fractures of children in 6-16 years of age

**DOI:** 10.4103/0019-5413.33876

**Published:** 2007

**Authors:** KC Saikia, SK Bhuyan, TD Bhattacharya, SP Saikia

**Affiliations:** Department of Orthopedics, Gawhati Medical College and Hospital, Guwahati -32, Assam, India

**Keywords:** Elastic titanium nailing, intramedullary nail, pediatric femoral fractures

## Abstract

**Background:**

Management of femoral diaphyseal fractures in the age group of 6-16 years is controversial. There has been a resurgence worldwide for operative fixation.

**Materials and Methods:**

Twenty-two children (18 boys, 4 girls) aged 6-16 years with recent (> 3 days) femoral diaphyseal fractures (20 closed, 2 open) were stabilized with Titanium Elastic Nail (TEN). These fractures were in proximal third (n=3), middle third (n=15) and in the distal third (n=4) 17 patients underwent surgery within seven days of their injury. The results were evaluated using Flynn's scoring criteria. Statistical analysis was done using Fischer's exact test.

**Results:**

All 22 patients were available for evaluation after a mean of 26 months (14-36 months) of followup. Radiological union in all cases were achieved in a mean time of 8.7 weeks. Full weight bearing was possible in a mean time of 8.8 weeks. Mean duration of hospital stay was 9.8 days. The results were excellent in 13 patients (59.0%), successful in six (27.2%) and poor in three patients (13.6%). All patients had early return to school.

**Conclusion:**

Intramedullary fixation titanium elastic nailing is an effective treatment of diaphyseal fractures of the femur in properly selected patients of the 6-16 years age group.

Femoral shaft fracture is an incapacitating pediatric injury.^1^,^2^ The treatment has traditionally been age-related, influenced by the type of injury, associated injuries and the location and type of the fracture. To a great extent, the treatment options vary according to the surgeon's preference.^3^ Because of rapid healing and spontaneous correction of angulation most of the femoral shaft fractures in children younger than six years of age can be treated conservatively.^4^,^5^ Above six years of age all such fractures, when treated nonoperatively could have, loss of reduction, malunion, intolerance and complications associated with plaster. Near the end of skeletal maturity accurate reduction is necessary as angular deformity is no longer correctable by growth.^6^ Availability of locked intramedullary nail has made the treatment of femoral shaft fractures in skeletally matured children well established. However, the best treatment between six and 16 years of age is a matter of debate.^7^ Since the last two decades, there has been a growing tendency towards a more operative approach in patents over six years of age.^3^,^6^,^8^ Titanium Elastic Nailing, which is variously known as Elastic Stable Intramedullary Nailing, has become the choice of stabilization in pediatric long bone fractures, particularly the femoral shaft fractures.^9^,^10^ The perceived advantage of this technique includes early union due to repeated micromotion at fracture site, respect for the physis, early mobilization, early weight bearing, scar acceptance, easy implant removal and high patient satisfaction rate.^1^,^3^,^9^,^11^

We report a prospective study with the objective of evaluating the role and efficacy of Titanium Elastic Nail in selected cases of femoral diaphyseal fractures in the 6-16 years age group.

## MATERIALS AND METHODS

**Figure 1:**
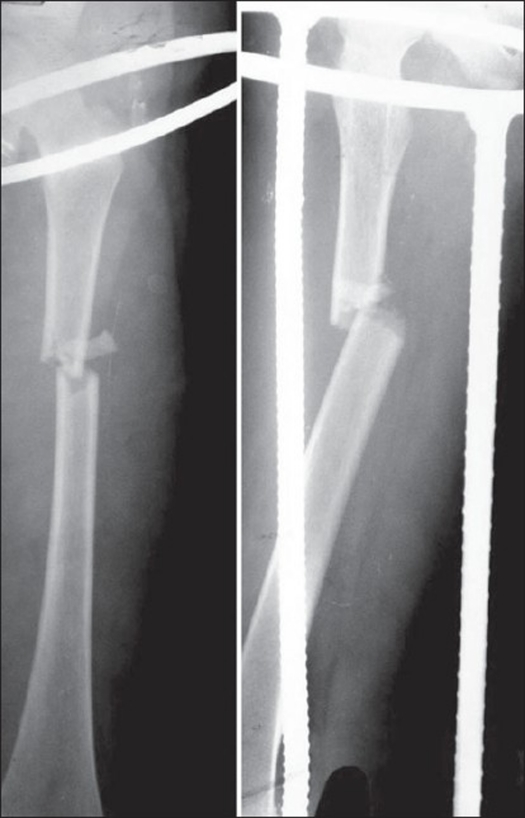
Preoperative x-ray of femur (AP and Lat View)

Twenty-two children (18 boys, four girls) in the age range of 6-16 years (average 10.8 years) with recent (> 3 days) femoral shaft fractures (20 closed, one Grade- I and one Grade- II compound) were stabilized with Titanium Elastic Nail (TEN), between January 2003 and February 2006. Most of the fractures were due to road traffic accidents (n=14, 63.6%). Right-sided involvement was seen in 12 cases (54.5%) and associated injuries were seen in five cases (22.7%). Three fractures were in the proximal third, 15 in the middle third and four were in the distal third. Twelve fractures were transverse, seven minimally comminuted (Winquist I) and three were short oblique [Figure 1]. Majority of the patients (n=17) underwent surgery within seven days of their injury. The surgery was performed under general anesthesia with the patient on the fracture table in supine position. Two Titanium Elastic Nails of identical diameter were used (18 of INOR and six of Synthes). Three cases required insertion of nails with different diameter because of the intraoperative difficulty in driving the second nail into the proximal fragment. The diameter of the individual nail was selected as per Flynn *et al*'s formula^1^ (Diameter of nail = Width of the narrowest point of the medullary canal on Anteroposterior and Lateral view × 0.4 mm) and intraoperative assessment. The diameter of the nail was chosen so that each nail occupies at least one-third to 40% of the medullary cavity. Fractures were reduced using fluoroscopic guidance. Fractures were inserted in retrograde fashion with medial and lateral incision 2.5-3.5 cm above the physis. The nails were prebent sufficiently so that apex of the bowed nails rested at the same level on the fracture site to ensure a good equal recoil force. Open reduction was required in three cases due to soft tissue interposition and failure to negotiate one nail to the proximal fragment. The nails were driven proximally so that both were divergent and the tips got anchored minimum 1 cm distal to the physis. Postoperatively patients were nursed in supine position with the operated leg elevated on a pillow. Long knee brace was used in three cases of distal third fractures, where fixation was not adequate. Patients were mobilized without weight bearing on the fifth to seventh day postoperatively. Partial weight bearing was started at three weeks and full weight bearing by six to eight weeks depending on the fracture configuration, callus response and associated injuries.

All patients were followed radiologically as well as clinically until fractures healed and for any complication. Statistical analysis was done using Fischer's exact test to evaluate

- The significance of association between the occurrence of skin site irritations and by long, untrimmed nail ends
- Association of angulation of fracture with smaller and mismatch nail diameter
- Outcome between patients < 10 years and >10 years.

**Figure 2:**
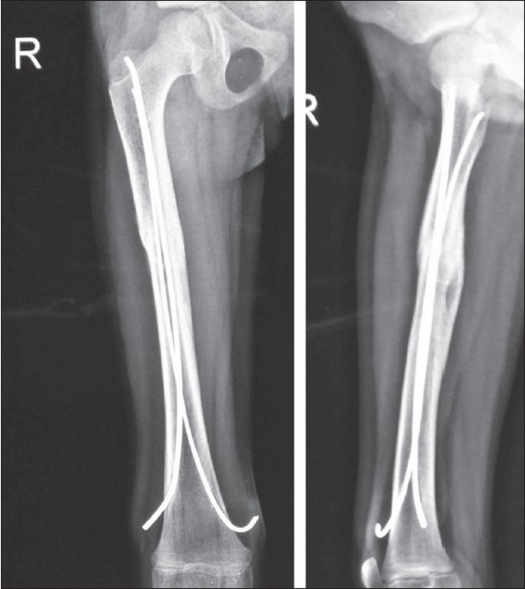
Six months postoperative (AP and Lat View) with solid union

**Figure 3:**
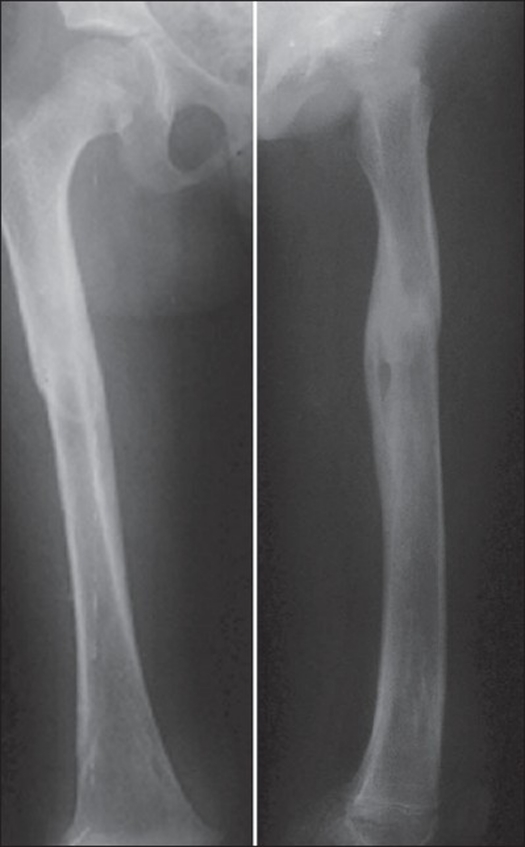
Two years followup (AP and Lat View)

The results were evaluated using Flynn *et al*'s scoring criteria for TEN^12^ [Table 1]. Nails were removed six to eight months post surgery when the fracture line was no longer visible radiologically [Figures 2, 3].

**Table 1:** The scoring criteria with Titanium Elastic Nails^12^

Limb length discrepancy	Excellent <1.0 cm	Successful <2.0 cm	Poor >2.0 cm
Sequence disorder	5°	10°	>10°
Pain	Absent	Absent	Present
Complication	Absent	Mild	Major complication and/or extended period for resolvable morbidity

## RESULTS

**Figure 4:**
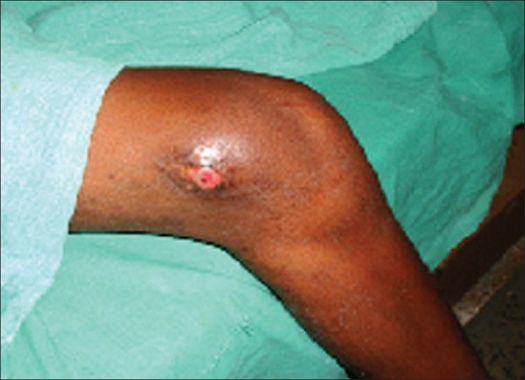
Clinical photograph showing entry site irritation at five months

The median duration of surgery was 70 min (50-120 min). The mean hospital stay was 9.8 days (8-21 days). A patient with head injury had to stay for a longer period (21 days). The hospital stay was dictated by associated injuries and the adequacy of fixation. All 22 patients were available for evaluation after a mean of 26 months (14-36 months) of followup. Radiological union was achieved in all cases in a mean time of 8.7 weeks (6-12 weeks). Full weight bearing was possible in a mean time of 8.8 weeks (6-12 weeks). The results were excellent in 13 patients (59.0%), successful in six (27.2%) and poor in three patients (13.6%) as per the scoring criteria for TEN by Flynn *et al*.^12^ Two patients had varus angulation (12° and 6° each) whereas one had valgus angulation (15°). Entry site irritation occurred in four patients [Figure 4]. Two patients had skin breakdown at entry site which led to superficial infection. The infection resolved with seven days oral course of cephalosporin. Limb lengthening of less than 1.5 cm was found in three cases both clinically as well as radiologically, which was clinically insignificant [Table 2]. One case required nail removal at fifth month due to wound breakdown at entry site. Results were better for children less than 10 years of age (*P* value-.0003). Leaving nail end long (>2 cm) and untrimmed was significantly associated with entry site irritation (*P* value.0001). Functional range of movement of knee was achieved in an average of 8.3 weeks (6-32 weeks).

**Table 2:** Clinical details of patients

Age yrs/sex	Nail diameter (mm)	Radiological union (wks)	Full weight bearing and return to school (wks)	Hospital days	Followup month	Remarks
8M	3.0	8	8	8	20	
10F	3.5	8	8	10	24	
13M	3.5	8	8	8	28	
19M	3.0	10	10	21	14	Head injury
6M	3.0	6	6	8	28	Limb lengthening-1.2 cm
12M	3.5	8	8	9	22	
14M	3.5	10	12	14	26	# Both bone forearm
13M	3.5	8	8	8	30	
7F	3.0	8	10	8	16	Limb lengthening-1.4 cm
11M	3.5	10	8	9	36	
10M	3.0	8	10	12	30	
13M	3.5	8	8	10	28	Varus angulation -12°
16F	4.0	12	12	12	30	Varus angulation-16°
15M	3.5	10	10	8	24	Valgus angulation-15°
6F	2.5	8	8	8	18	Limb lengthening-1.3 cm
10M	3.5	10	8	8	22	
12M	3.5	10	10	10	26	
7M	3.0	6	8	8	30	
12M	3.5	10	10	12	34	
16M	3.5	8	8	8	30	
9F	3.0	8	8	8	28	
11M	3.5	10	10	9	32	

## DISCUSSION

Although femoral shaft fractures constitute fewer than 2% of all pediatric fractures, the choice of treatment has remained a constant challenge to the orthopedics fraternity. Until recently conservative treatment was the preferred method for the treatment of diaphyseal fractures in children and young adolescents. However, to avoid the effects of prolonged immobilization, to reduce the loss of school days and for better nursing care, the operative approach has been gaining popularity for the last two decades. Plate osteosynthesis is still widely used. It is associated with a large exposure, relatively longer duration of immobilization and the risks of delayed union, infection and a large dissection for plate removal.^13^,^14^ The external fixator provides good stability and early mobilization, but is associated with the risk of pin tract infections and it takes a longer time for weight bearing.^15^,^16^ Intramedullary K-wire fixation has also been used for pediatric femoral fracture. But stability and fracture angulation is a disadvantage to be taken care of. Interlocking nail is ideal for skeletally matured children. Reports of avascular necrosis of femoral head, coxa valga have been reported with interlocking nail when attempted in skeletally immature patients.^18^,^19^ However there have been proponents for using interlocking nail in the 11-16 years of age group, avoiding the pyriformis fossa as entry site, with good results.^20^

Titanium elastic nail seems advantageous over other surgical methods particularly in this age group because it is simple, is a load-sharing internal splint that doesn't violate open physis, allows early mobilization and maintains alignment. Micromotion conferred by the elasticity of the fixation promotes faster external bridging callus formation. The periosteum is not disturbed and being a closed procedure there is no disturbance of the fracture hematoma, thereby less risk of infection. Flynn *et al*. found TEN advantageous over hip spica in treatment of femoral shaft fractures in children.^7^ Buechsenschuetz *et al*, documented titanium nail superior in terms of union, scar acceptance and overall patient satisfaction compared to traction and casting.^21^ Ligier *et al*. treated 123 femoral shaft fractures with elastic stable intramedullary nail. All fractures united. Thirteen children developed entry site irritation.^22^ Similarly, Narayanan *et al.* found good outcome in 79 femoral fractures stabilized with TEN.^3^

There is no comparative study regarding the efficacy of Ender Nail, Rush Nail or Titanium Elastic Nail. All the nails give good results. Ender Nail and Rush Nail have poor rotational stability and require multiple nails to achieve good fixation. Moreover, Ender Nail is not elastic and flexible enough for pediatric fractures as stated by Ligier.^22^ Heinrich *et al*. observed good results in 78 femoral fractures treated with Ender Nail.^23^

Fracture geometry and the location is an important determinant for selection of surgical techniques. Transverse, short oblique and minimally comminuted fractures are suitable for TEN as stated by Flynn *et al*.^1^ Narayanan *et al*.^3^ stated that transverse, short oblique, short spiral fractures with minimum comminution in the 5-12 years age group were the best indications for TEN. Lascombes *et al*.^24^ stated that TEN could be indicated in all femoral diaphyseal fractures of children above six years of age till epiphysis closed except severe Type III open fractures. Titanium elastic nail does not provide adequate stability in comminuted, long oblique or spiral fractures. Even if it is contemplated, postoperative immobilization becomes essential. Appropriate alternatives other than titanium elastic nail should be considered in such circumstances.

The most common complication of Titanium elastic nail is entry site irritation and pain.^3^,^12^ Other complications include limb length discrepancy, angulation of fracture, refractures and infection. Entry site irritation in our series was seen in four cases. We found that entry site irritation was significantly associated with long and prominent nail end (>2 cm). Similarly smaller and mismatch nail diameter that was incidentally used in three cases was associated with increased incidence of varus/valgus angulation, which conforms to the finding by Narayanan *et al* in their series. All these findings were statistically significant.

## CONCLUSIONS

The titanium elastic nailing is an effective and viable treatment option in selected cases of femoral diaphyseal fractures in the 6-16 years age group.
